# Implementation fidelity of intravenous ferric carboxymaltose administration for iron deficiency anaemia in pregnancy: a mixed-methods study nested in a clinical trial in Nigeria

**DOI:** 10.1186/s43058-024-00609-5

**Published:** 2024-07-23

**Authors:** Opeyemi R. Akinajo, Kristi Sidney Annerstedt, Aduragbemi Banke-Thomas, Chisom Obi‑Jeff, Nadia A. Sam-Agudu, Ochuwa A. Babah, Mobolanle R. Balogun, Lenka Beňová, Bosede Bukola Afolabi

**Affiliations:** 1 Department of Obstetrics and Gynaecology, Lagos University Teaching Hospital, Lagos, Idi-Araba Nigeria; 2 Department of Global Public Health, Karolinska Institutet, Stockholm, Sweden; 3 Department of Public Health, Institute of Tropical Medicine, Antwerp, Belgium; 4 Department of Obstetrics and Gynaecology, Faculty of Clinical Sciences, College of Medicine, University of Lagos, Lagos, Idi-Araba Nigeria; 5 Centre for Clinical Trials and Implementation Science (CCTRIS), College of Medicine, University of Lagos, Lagos, Idi-Araba Nigeria; 6 Maternal, Adolescent, Reproductive and Child Health (MARCH), Centre, London School of Hygiene and Tropical Medicine, London, UK; 7 Brooks Insights Limited, Abuja, FCT Nigeria; 8 Department of Infectious Disease Epidemiology, London School of Hygiene and Tropical Medicine, London, UK; 9 International Research Center of Excellence, Institute of Human Virology Nigeria, Abuja, Nigeria; 10 Global Pediatrics Program and Division of Infectious Diseases, Department of Pediatrics, University of Minnesota Medical School, Minneapolis, Minnesota, USA; 11 Department of Community Health & Primary Care, College of Medicine, University of Lagos, Lagos, Nigeria

**Keywords:** Anaemia, Iron deficiency anaemia, Pregnancy, Fidelity, Intravenous iron, Implementation science, Nigeria, Sub-Saharan Africa

## Abstract

**Background:**

Iron deficiency anaemia is common among pregnant women in Nigeria. The standard treatment is oral iron therapy, which can be sub-optimal due to side effects. Intravenous ferric carboxymaltose (FCM) is an evidenced-based alternative treatment with a more favourable side effect profile requiring administration according to a standardized protocol. In this study, we assessed the fidelity of administering a single dose of FCM according to protocol and identified factors influencing implementation fidelity.

**Methods:**

We used a mixed-method approach with a sequential explanatory design nested in a clinical trial across 11 facilities in Lagos and Kano States, Nigeria. Guided by a conceptual framework of implementation fidelity, we quantitatively assessed adherence to protocol by directly observing every alternate FCM administration, using an intervention procedure checklist, and compared median adherence by facility and state. Qualitative fidelity assessment was conducted via in-depth interviews with 14 skilled health personnel (SHP) from nine purposively selected health facilities, using a semi-structured interview guide. We analyzed quantitative data using descriptive and inferential statistics in Stata and used thematic analysis to analyze the transcribed interviews in NVivo.

**Results:**

A total of 254 FCM administrations were observed across the 11 study sites, with the majority in secondary (63%), followed by primary healthcare facilities (PHCs) (30%). Overall, adherence to FCM administration as per protocol was moderate (63%) and varied depending on facility level. The lowest level of adherence was observed in PHCs (36%). Median, adherence level showed significant differences by facility level (*p* = 0.001) but not by state (*p* = 0.889). Teamwork and availability of protocols are facilitation strategies that contributed to high fidelity. However, institutional/ logistical barriers are contextual factors that influenced the varied fidelity levels observed in some facilities.

**Conclusions:**

Collaborative teams and access to operating protocols resulted in high fidelity in some facilities. However, in some PHCs, fidelity to FCM was low due to contextual factors and intervention complexities, thereby influencing the quality of delivery. In Nigeria, scale-up of FCM will require attention to staff strength, teamwork and availability of administration protocols, in order to optimize its impact on anaemia in pregnancy.

**Supplementary Information:**

The online version contains supplementary material available at 10.1186/s43058-024-00609-5.

Contributions to the literature

- Intravenous iron (IV) is a well-established evidence-based intervention for iron deficiency anaemia (IDA), but not for several African countries. This study is the first in Nigeria to investigate the adherence of skilled health personnel to IV iron administration for treating IDA in pregnancy.
- Our study provides new insights into implementing this intervention in an African setting and offers strong evidence of the impact of selected implementation strategies on ensuring the fidelity of IV iron administration across various health facilities.
- Furthermore, it highlights the specific challenges that need to be addressed to scale up this intervention successfully in our setting.

## Background

Iron deficiency anaemia (IDA) is the leading cause of anaemia in pregnancy (AIP) globally [1], contributing to up to 25–45% of AIP in Nigeria [2]. This condition can be categorized as mild, with a haemoglobin (Hb) concentration of 10–10.9 g/dl, moderate with Hb of 7.0–9.9 g/dl, and severe with Hb < 7.0 g/dl [3]. To reduce the high risk of fetomaternal morbidity and mortality [2, 4] associated with this condition in low- and middle-income countries (LMICs), the World Health Organization recommends daily oral iron as prophylaxis and therapeutic using low and higher doses, respectively [3, 5]. However, higher doses are associated with significant gastrointestinal adverse events [6, 7], which result in intolerance and poor adherence in up to 70% of pregnant women [8–11].

Ferric carboxymaltose (FCM), is a dextran-free intravenous (IV) iron therapy which is typically used when oral iron therapy is ineffective either from poor adherence, lack of tolerance, lack of absorption, side effect or a need for quick correction of low haemoglobin (Hb) levels [6]. FCM is widely used as an alternative for treating moderate to severe IDA in pregnancy, especially in high-income countries (HICs) [6, 12]. However, it has only recently been the subject of clinical trials in LMICs, including Nigeria and Malawi [13, 14]. Despite the positive results of these trials, the nature of administration of IV FCM, as well as the timing and severity of adverse events related to it, are very different to that of oral iron [6]. As such, there is a need to adequately` train skilled health personnel (SHP), i.e., doctors and nurses, to safely administer this intervention and manage any reactions that may occur during or after administration [6, 15, 16].

Fidelity can be described as how closely an intervention like FCM is administered according to the protocol [17]. Fidelity is a crucial outcome for researchers planning on translating evidence-based interventions (EBIs) into day-to-day practice [17]. High fidelity can enable the maintenance of the intended effect of EBIs, even if delivered in a different context [18]. Additionally, fidelity assessment helps to distinguish between the EBI’s actual effects and variations in its implementation [19]. Therefore, for any EBI like FCM to be successfully and safely administered, evaluating the adherence to the protocol, the dose, and the quality of intervention delivery is crucial [17].

While there is a general recommendation for IV iron as a treatment option for AIP, there are currently no established guidelines for administering IV iron to pregnant women in Nigeria [20]. It is important to support the IV iron recommendation with feasible standardized protocols for safe and accurate administration. Healthcare facilities that attain high implementation fidelity might develop the capacity to adopt and integrate FCM into routine practice.

However, there is insufficient evidence on the implementation fidelity of this EBI in LMICs [21]. To address this evidence gap, the objective of this study was to assess the fidelity of SHP in administering FCM safely to treat moderate to severe IDA in pregnancy and to understand factors which influence implementation fidelity within the setting of a clinical trial in Nigeria.

## Methods

### Study design and setting

This mixed-method study of sequential explanatory design consisted of two phases: a quantitative followed by a qualitative phase [22]. In the quantitative phase, data on each component of the administration of FCM were collected and analyzed. Findings from this analysis informed the subsequent qualitative sampling, data collection, and analysis. This study was nested in the context of an open-label hybrid study of IV versus oral iron for iron deficiency anaemia in pregnant women in Nigeria (IVON) [23]. We used the Standards for Reporting Implementation Studies (StaRI) checklist to report our findings (Additional file I) [24].

This study was conducted in IVON trial sites in the two most populous States in Nigeria: Kano and Lagos [25]. Nigeria is a country of > 200 million inhabitants, with an estimated seven million births each year [26] and a high maternal mortality ratio of 917 per 100,000 live births [27]. These two states are situated in the North-Western (Kano) and South-Western (Lagos) parts of Nigeria [26] with varying antenatal care utilization rates (Kano: 73% and Lagos: 90%) [28]. The prevalence of AIP in Kano is approximately 47%, while in Lagos, it is 50% [26]. We conducted the study across the three levels of the healthcare system namely primary, secondary, and tertiary [25]. Primary healthcare (PHC) provides basic maternal healthcare services at the community level, while secondary and tertiary facilities provide comprehensive and advanced maternal healthcare [29]. These facilities included two PHCs in Lagos and three PHCs in Kano, and two secondary and one tertiary facilities in each state.

### Conceptual framework

We adapted the modified Carroll et al. conceptual framework for implementation fidelity (CFIF) to assess the implementation fidelity, henceforth referred to as fidelity, of FCM administration across different levels of healthcare facilities [30–32]. According to CFIF, implementation fidelity is the measurement of adherence (e.g., the extent to which those delivering an intervention adhere to its original design) which is influenced by factors including facilitation strategies, intervention complexity, delivery quality, and participant responsiveness. We measured adherence quantitatively using its subcategories (content, coverage, dose, and duration) and evaluated qualitatively the influencing factors. The different fidelity components and measurement methods are defined in more detail in Table 1.

**Table 1:** Elements of implementation fidelity for FCM administration adapted from Carroll et al. elements and modified by Hasson Henna

Elements and sub-elements of implementation fidelity	Definition	Method/measurement for Implementation fidelity
***1. Adherence***	*The degree to which FCM is delivered as intended*	*Quantitative*
Content	The necessary steps (active ingredients) in administering FCM	Number of FCM administration with complete adherence to the active ingredient of FCM administration (the essential (*n* = 10) components) / total number of FCM administration observed (*n* = 254)
Coverage	The proportion of eligible participants who actually received FCM administration	Total number of FCM administration observed/ Number of participants eligible for FCM administration
Dose	Amount of FCM administered as intended	Number of FCM administrations that adhered to the right amount (20 mg/kg) / total number of FCM observed
Duration	Regularity of the time to monitor for adverse events post-FCM administration	Number of FCM administered that adhered to the right duration of time (30 min) for monitoring post-administration / total number of FCM observed
***2. Influencers of fidelity***	*Factors that influence the degree of implementation fidelity*	*Qualitative*
Intervention complexities	The comprehensiveness of FCM description and the complexity associated with its administration	In-depth interviews (IDI)
Facilitation strategies	Strategies provided to optimise and standardise implementation fidelity,	
Quality of delivery	The degree to which FCM is delivered in a way appropriate to achieving its desired outcome	
Participant’s responsiveness	The acceptance and engagement of the SHP to FCM administration	
Context	Structural, cultural, organisational / group factors that influence FCM administration	

*SHP* Skilled health personnel, *FCM* ferric carboxymaltose

### Implementation strategies

The SHP who administered FCM were skilled research nurses (employed by the trial), facility-based nurses who assisted the research nurses as needed, and medical doctors specializing in fetomaternal care. The trial organized a theoretical and practical training session to ensure that all SHP could administer FCM and perform basic life support. The trial also provided treatment protocols/standard operating procedure (SOP), counselling guidelines and charts to simplify the calculation of the dose of FCM. An algorithm was developed to facilitate step-by-step administration processes [33], which was further used to train the SHP and ensure consistency in FCM administration across all trial facilities (Additional file II). Emergency drugs (e.g. adrenaline and hydrocortisone) and resuscitation materials were provided by the IVON research team to each facility to manage anaphylactic reactions in the unlikely event that they occurred. During the implementation phase of the 16-month trial, six monthly refresher training sessions (four in all), quarterly monitoring, and monthly feedback were provided by IVON to all SHP involved in the trial in all 11 facilities (Table 2).

**Table 2:** Illustrating implementation strategies that facilitated implementation outcome

Phase	Implementation strategies
Pre-implementation phase	Healthcare providers training on basic life support
	Baseline and periodic healthcare providers training on the safe administration of IV iron
	Provision of treatment protocol, guidelines, counselling charts and algorithm
	Provision of materials and equipment to promote adaptability
Implementation phase	Regular monitoring of progress and continuous engagement to improve the quality of care
	Monthly feedback to the SHP

## Data collection tools and sources

To assess the fidelity of FCM administration (Table 3), the following four tools were used:

1. Intervention procedure checklist**:** A checklist was developed to assess the adherence (content) of the FCM administration (Additional file III). The IVON trial treatment manual, FCM administration package insert, and a literature search on the safe administration of IV iron guided the initial development of the checklist [34–37]. The checklist was then divided into smaller, sequenced steps, resulting in a 12-item (components) standardized intervention procedure checklist. Subsequently, 10 essential components critical to safely administering IV iron were identified. The first component included nine sub-components comprised of resuscitation medications and materials. This checklist was confirmed through SHP and core research team feedback, ensuring content validity.
2. FCM administration chart: We used an intervention chart to track FCM administration details, including participant identifiers (study ID and study site name), dose, vital sign checks (temperature, oxygen saturation, respiratory rate, pulse rate and blood pressure), and administration duration. The intervention chart was developed to record the details of each administration procedure including the starting and ending time of the administration, the cadre of SHP involved, and the intervals for vital signs checks. These vital signs were conducted before administration, two minutes into the administration, immediately after, and 30 min post-administration. Additionally, the chart was used to record the clinical state of the participants throughout the administration process and at the time of discharge.
3. Enrolment data: To confirm the proportion of eligible participants who received FCM administration, we measured coverage using the IVON trial enrolment data on Research Electronic Data Capture (REDCap) [38, 39].
4. Topic guide: Using the CFIF, a semi-structured topic guide with four sections was developed to understand factors influencing adherence in this study. This guide was utilized for qualitative data collection via in-depth interviews (IDIs) among purposively selected SHP.

**Table 3:** Illustrating data collection sources for implementation fidelity elements

Data sources	Adherence				Potential Influencers				
	**Content**	**Coverage**	**Dose**	**Duration**	**IC**	**FS**	**QA**	**PR**	**C**
**Quantitative**									
Intervention procedure checklist	X								
FCM administration chart	X	X	X	X					
Enrolment data on Redcap		X							
**Qualitative**									
In-depth interviews with SHP					X	X	X	X	X

*IC* intervention complexities, *FS* facilitation strategies, *QA* Quality of FCM administration, *PR* participants responsiveness, *C* context, *SHP* Skilled Health Personnel

## Recruitment procedure

In the IVON trial, all 11 facilities were involved in the implementation of FCM. For this study, we used a systematic sampling technique (every alternate FCM administration) to select FCM administration to assess adherence to the study protocol. The target sample size was 264, out of which the first ten administrations were used to pilot the intervention procedure checklist. A total of 254 observations of FCM administrations, ranging from nine to 58 per facility, were included in the analysis.

Qualitatively, to gain insight into the factors influencing adherence to FCM administration protocols, we purposively selected facilities with low and high adherence and high antenatal patient attendance (compared to the number of available SHP). To determine data sufficiency, we relied on the concept of information power [40] based on the study objective, the specificity of the sample, and the high-quality input from different perspectives [24].

## Data collection

### Quantitative phase

We assessed adherence to content through direct non-participant observation with the intervention procedure checklist. To do this, we trained seven neutral research assistants to use the checklist, and to document and interpret observations accurately. Each element of adherence was rated by the observers as done, partially done, or not done. Data were collected between August 9, 2021, and December 29, 2022, and entered directly into REDCap (Vanderbilt University, Nashville, Tennessee, US) using an electronic device (tablet).

### Qualitative phase

We developed a four-section interview guide based on the findings from the quantitative aspect of this study and insights from previous studies on barriers to administering FCM (Additional file IV) [15, 16]. We piloted the guide before data collection and made necessary adjustments based on the responses of the SHPs.

The first author (ORA) and two other interviewers (research assistants) trained in qualitative data collection methods conducted the IDIs with SHP in selected healthcare facilities. We chose IDIs as they provided a practical opportunity for one-on-one discussions with the SHP involved in the intervention [41, 42]. IDIs were conducted over the phone at the convenience of the SHP in those facilities to create a comfortable and non-judgemental environment. We interviewed 14 SHP purposively selected from nine facilities between May and October 2023 in English between 40 and 90 min. We ensured the accuracy and clarity of intended meanings by repeating responses to participants. All interviews were audio-recorded and transcribed verbatim.

## Data analysis

### Quantitative data analysis

The number of FCM observations per facility level was presented using descriptive statistics in frequencies and percentages. Percentages for the sub-elements of adherence (content, coverage, dose, and duration) were presented, including the 10 essential components for content. The nine sub-components of the first component were added and then divided by nine to create a proportion completed. *Adherence per protocol* was also calculated as a proportion, and this was defined as *all* 18 essential components from the intervention procedure checklist (content) in addition to dose and duration.

The level of adherence per facility level was examined using Pearson’s Chi-square test. We utilized the Shapiro–Wilk test to test for normality distribution. Median and interquartile ranges were used to present adherence levels for non-normally distributed data by components.

The adherence levels per facility level for each sub-element were categorized as high (between 80–100%), moderate (between 51–79%), and low (between 0–50%) based on insights from published studies [43, 44]. The Kruskal–Wallis test was utilized to identify significant differences between adherence levels per facility level and state, and a *p*-value of less than 0.05 was considered significant. A post hoc test was conducted utilizing the Bonferroni-Dunn test to determine the exact facility type with a statistically significant difference. With further analysis, we identified and selected healthcare facilities purposively for qualitative data collection. We analyzed our data using Stata version 17.0 statistical software (StataCorp LLC, College Station, Texas, US).

### Qualitative data analysis

The modified CFIF framework informed our analysis, which focused on implementation fidelity [30–32]. All transcribed interviews were reviewed for accuracy and completeness and analyzed using thematic analysis [45, 46]. ORA and COJ, two qualitatively trained researchers, coded a subset of the transcript independently using a deductive approach to create a codebook, which was applied to the rest of the dataset. Next, we developed, reviewed, and refined our themes and sub-themes to accurately reflect the dataset's meaning. The analysis was conducted with the aid of a computer-assisted qualitative data analysis software, NVivo 12 Plus (QSR International, Memphis, Tennessee, US).

## Data integration

We prioritized the initial quantitative findings and used the qualitative findings to provide further explanation. Ultimately, we integrated quantitative and qualitative results in the interpretation phase of the findings [47]. Data from FCM administration observation and IDIs with SHP were quantitatively and qualitatively triangulated to ascertain convergence. Convergence was defined as general agreement between data sets.

## Ethical considerations

Ethical approval was obtained from the National Health Research Ethics Committee of Nigeria (NHREC/01/01/2007- 17/01/2021), Health Research and Ethics Committees of the Lagos University Teaching Hospital (ADM/DCST/HREC/APP/3971) and Aminu Kano Teaching Hospital, Kano State (NHREC/28/01/2020/AKTH/EC/2955).

Social approvals were obtained from Lagos State Health Service Commission (LSHSC/2222/VOLIII), Lagos State Primary Health Care Board (LS/PHCB/MS/1128/VOL.VII/100) and Ministry of Health, Kano State (MOH/Off/797/T.1/2102). We obtained verbal consent from SHP before each phase of the study. Patient safety was ensured by avoiding interruptions during the observation process. Audio recordings before data collection began only after obtaining verbal permission from the SHP.

## Results

### Quantitative findings

#### Characteristics of FCM administration observations

The 254 observed administrations of FCM to pregnant women in the 11 trial facilities were conducted by 42 unique SHP. Mirroring the distribution of participants in the clinical trial, the majority of the observations were conducted at secondary healthcare facilities (63%), followed by PHCs (30%), and 7% in tertiary facilities. One hundred and twenty-four FCM administrations were observed in Lagos state (49%) and 130 (51%) in Kano state.

#### Adherence in implementation fidelity

There was a high level of adherence to each of the ten essential components (content). Ensuring the availability of FCM had the highest adherence (100%) and ensuring the availability of resuscitation materials such as oxygen masks had the lowest adherence (73%). When comparing the median adherence levels among the states, we found no significant difference (*p* = 0.889). However, the detailed results are not presented in Table 4 below. The median adherence level for PHC was 97.8 (IQR = 81.1,100), 100 in secondary (IQR = 100,100), and 100 in tertiary facilities (IQR = 100,100) (Table 4). The level of adherence per facility level was significant *(p* < 0.001), with median adherence levels which statistically differed by facility level (*p* = 0.001). There was a statistically significant difference in adherence levels between PHC and secondary facilities (*p* < 0.001), and between PHC and tertiary facilities (*p* < 0.001), but no difference between secondary and tertiary facilities (*p* = 0.394). All eligible participants (coverage measure) received FCM (100%). The level of adherence to dose (99.6%) and duration (95.3%) were high.

**Table 4:** Adherence for content, dose, and duration by facility level and overall (*n *= 254 FCM administrations)

Content	Primary (five facilities) (*n* = 75) (%)	Secondary (four facilities) (*n* = 160) (%)	Tertiary (two facilities) (*n* = 19) (%)	All facilities (*n* = 254) (%)
1. Ensure the availability of the following resuscitation medications and materials at the point of administration				
i. Adrenaline	61 (81.3)	144 (90.0)	17(89.4)	**222(87.4)**
ii. IV Chlorpheniramine	57 (76.0)	144 (90.0)	16 (84.2)	**217 (85.4)**
iii. IV Ranitidine	61 (81.3)	144 (90.0)	17 (89.5)	**222 (87.4)**
iv. Oral Ranitidine	55 (73.3)	141 (88.1)	16 (84.2)	**212 (83.5)**
v. IV hydrocortisone	61 (81.3)	154 (96.2)	17 (89.5)	**232 (91.3)**
vi. 500mls/1L bottle normal saline	61 (81.3)	156 (97.5)	17 (89.5)	**234 (92.1)**
vii. Intravenous fluid giving set	63 (84.0)	160(100.0)	18 (94.7)	**241 (94.9)**
viii. Oxygen mask	40 (53.3)	131 (81.9)	16 (84.2)	**187 (73.6)**
ix. Oxygen cylinder	38 (50.7)	158 (98.8)	17 (89.5)	**213 (83.9)**
**Total median score for component 1 (IQR)**	**88.9 (66.7–100.0)**	**100.0 (100.0,100.0)**	**100.0 (100.0,100.0)**	**100.0 (88.9–100.0)**
2. Ensure availability of FCM	75 (100.0)	160(100.0)	19(100.0)	**254 (100.0)**
3. Patient counselling	62 (82.7)	160(100.0)	19(100.0)	**241 (94.9)**
4. Confirm patient’s randomization group is FCM	75 (100.0)	160(100.0)	19(100.0)	**254 (100.0)**
5. Obtain verbal consent for FCM administration from participants	65 (86.7)	159 (99.4)	19 (100.0)	**243 (95.6)**
6. Perform baseline vital signs	65 (86.7)	159 (99.4)	19 (100.0)	**243 (95.6)**
7. Calculation of individual dose at 20 mg/Kg body weight up to a maximum dose of 1000 mg	75 (100.0)	156 (97.5)	19 (100.0)	**250 (98.4)**
8. Set up the administration at a low flow rate	70 (93.3)	158 (98.8)	19 (100.0)	**247 (97.3)**
9. Observe the patient closely and monitor vital signs for the first 2 min	59 (78.7)	159 (99.4)	19 (100.0)	**237(93.3)**
10. Post-administration vital signs check	54 (72.0)	160(100.0)	19 (100.0)	**233 (91.7)**
**Total median score for content (IQR)**	**97.8 (81.1–100.0)**	**100 (100.0, 100.0)**	**100 (100.0, 100.0)**	**100.0 (97.8–100.0)**
**Dose**				
Withdraw the equivalent volume of the calculated dose	75 (100.0)	159(99.4)	19 (100.0)	**253 (99.6)**
**Duration**				
Patient is observed for adverse effects for at least 30 min following administration	69 (92.0)	155 (96.9)	18 (94.7)	**242 (95.3)**

*IQR* Interquartile range

#### Adherence level to protocol

**Fig. 1:**
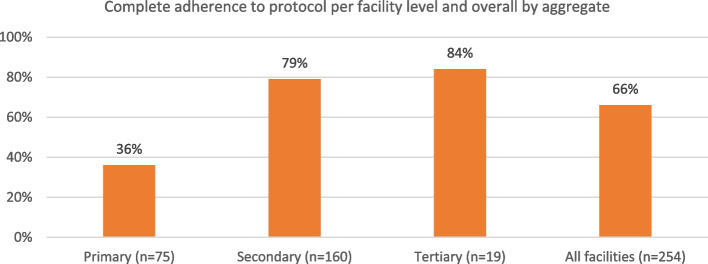
Level of adherence to protocol per facility level and overall (n: no of FCM administrations per facility)

The level of adherence to protocol was highest in tertiary facilities (84.2%) and lowest in PHCs (36.0%) (*p* < 0.001). Overall, the level of complete adherence to protocol was moderate (66.5%) (Fig. 1).

Further data stratification by facility levels and States revealed that ensuring the availability of resuscitation drugs and materials at the point of administration (component 1) as the most affected component with incomplete adherence to content across all three healthcare facility levels (Table 4). Adherence levels to this component ranged from 37.2% to 100.0% across the facility levels in both states, with statistically significant differences between PHC and secondary facilities (*p* < 0.001) and PHC and tertiary facilities (*p* < 0.001) (Table 5). Components 3 (patient counselling), 6 (perform baseline vital signs) and 10 (post-administration vital signs check) were the next most common contributors to incomplete adherence (Table 5).

**Table 5:** Essential components with the lowest adherence level by facility type and state

Element of adherence	The essential components with the lowest adherence level by facility type per state (%)			
**Component**	**State**	**Primary Healthcare Centres**	**Secondary Healthcare Centres**	**Tertiary Healthcare Centres**
**Content**				
1. Ensure the availability of resuscitation drugs and materials at the point of administration	Lagos	90.0 (PHC #1& 2)	80.0 (SHF #1)	95.0
	Kano	37.0 (PHC #3)	95.0 (SHF #1)	80.0
3. Patient Counselling	Lagos	90.0 (PHC #1 & 2)	100.0	100.0
	Kano	80.0 (PHC #2)	100.0	100.0
6. Perform baseline vital signs	Lagos	100.0	100.0	100.0
	Kano	70.0 (PHC #3)	95.0 (SHF #1)	100.0
10. Post-administration vital signs check	Lagos	100.0	100.0	100.0
	Kano	50.0 (PHC #2)	100.0	100.0

#1,2,3 represents the nomenclature of the healthcare level in each state

Four facilities had varied adherence levels (from low to high): PHC facilities #2 and #3 in Kano, secondary facility #1 in Lagos and tertiary facility in Kano (PHC #3 – > PHC #2 – > SHF #1 – > TF). In contrast, PHC facilities #1 (93.8%) and #2 (95.0%) in Lagos, secondary facility #2 in Lagos (100%) and secondary facility #2 in Kano (99.7%) had the highest adherence levels**.**

## Qualitative findings

Three main themes primarily influenced the level of adherence to FCM administration (content) in this study namely teamwork is key to sustained high fidelity, readily available charts and protocols are needed for high fidelity and institutional/logistical barriers negatively influenced fidelity. The third theme was further divided into three sub-themes to describe the institutional barriers as influencing factors of fidelity. These themes and sub-themes were mapped to all but one of the elements of the modified CFIF (Table 6).

**Table 6:** Themes and sub-themes mapped into five factors that influenced the implementation fidelity of FCM administration using the modified CFIF

**Themes**	**Sub-themes**	**CFIF elements**
1. Teamwork is key to sustained high fidelity	-	Facilitation strategies Quality of delivery
2. Readily available charts and protocols are needed for high fidelity	-	Facilitation strategies Quality of delivery
3. Institutional/logistical barriers negatively influenced fidelity	3.1 Ensuring the availability of resuscitation materials at the point of administration is a challenge	Intervention complexities Facilitation strategies Context Participant’s responsiveness Quality of delivery
	3.2 High SHP workload, necessitated expedited conduct of counselling and baseline observation	
	3.3 Prolonged patient waiting time influenced post administration observation	

## Teamwork is key to sustained high fidelity

For most SHP, administering FCM to pregnant women was a simple procedure that closely followed standard clinical practice. However, to administer FCM as intended, teamwork was deemed to be of utmost importance. According to some SHP who had sustained high fidelity levels throughout the trial, teamwork was critical to administer FCM as intended. They reported encountering many challenges that could have negatively influenced FCM level. Nonetheless, they overcame these challenges by effectively working as a team despite dealing with high patient flow and a limited workforce.

“*I have very hard-working research nurses… they have tried, so it is teamwork. Every one of us participated very well and put in our best, which is why you see such improvement, such performance*”. ***Medical Officer, Female, Secondary Healthcare Facility #2, Kano***

One of the research nurses in PHC #2 Lagos emphasized the importance of teamwork when administering FCM, especially when there is more than one administration to be made. She shared her experience of a time when she had to administer FCM to three eligible pregnant women with a challenge to monitor them at once. However, the administration process became much easier when the facility nurse joined her to assist. According to her, they attended to one pregnant woman at a time and then moved on to the next until all three pregnant women had received their FCM. This approach enabled them to complete the administration process as intended.

## Readily available charts and protocols needed for high fidelity

According to SHP in highly adherent facilities, the readily availability of charts and protocols contributed to their high performance. Most influential was the availability of charts, which were pasted on the wall for easy access when needed.

“*You know, as a team, we decided to follow a step-by-step method, so I’m always looking at the chart on the wall, and that was how I was doing until I became so familiar with it. Even after that, I visit the chart whenever I want to administer the IV iron (FCM”*. ***Research Nurse, Female, Secondary Healthcare Facility #2, Lagos***

## Institutional/logistical barriers negatively influenced fidelity

Institutional/logistical barriers negatively influenced the adherence of most SHP to components 1,3, 6 and 10 as intended.

### Ensuring the availability of resuscitation materials at the point of administration

Component 1 (availability of resuscitation materials at the point of administration) was not delivered as intended at several sites. Non-proximity between the storage area for resuscitation drugs/ materials and where FCM was administered created an issue. They overcame this challenge after discussions with the IVON research team, when storage space was provided within the FCM administration area.

*Space was why ensuring the availability of resuscitation materials at the point of administration was difficult to implement as planned at the initial phase of the trial. I mentioned space because where we used it was not totally ours. We didn't get a place to keep the resuscitating drugs…so we had to keep them on another floor and go up and down the stairs to bring them when needed…so it was not easy at all because it was only me at that initial phase… but with time they provided us with a cupboard to use. And we started having our things close as instructed, making the administration more straightforward." ****Research Nurse, Female, Secondary Healthcare Facility #1, Lagos***

For some SHP in the tertiary and PHC facility levels, component 1 was not adhered to as intended because they were aware that the resuscitation drugs and materials were readily accessible and stored in a secured place. Therefore, they felt it unnecessary to check the availability of these drugs at the point of every administration as per the protocol.

### High HCP workload expedited conduct of counselling and baseline observations

Even though the SHP were adequately trained to counsel the pregnant women on the process of FCM administration just before the procedure, the high SHP workload in the facilities influenced their complete adherence to this component. Therefore, counselling pregnant women (component 3) as intended became challenging. According to a nurse in PHC #2, Lagos, the one-on-one counselling before FCM administration might be rushed through due to the pressure to administer FCM to many eligible pregnant women at the same time. From the perspective of another SHP, challenges to adhering to this component as intended occurred when:

“… *there are so many patients to administer FCM to at the same time, and usually, in such instances, the patients want to go home on time, and the SHP are also trying their best not to delay them, so further verbal counselling may not be very adequate, you might actually miss some things*…”*** Medical officer, Female, Primary Healthcare Facility #1, Lagos***

Furthermore, the high SHP workload was used to explain why component 6 (to perform baseline vital signs check) was not completely adhered to. Although most SHP performed this task, a few found it challenging, citing forgetfulness and heavy workload as the reasons why. However, SHP at a high-fidelity facility in Kano overcame this challenge by complementing their workforce's capacity with the number of patients receiving FCM daily. This strategy resulted in decreased waiting time, agitation, and anxiety for both SHP and pregnant women.

### Prolonged patient waiting time influenced post administration observation

According to some SHP, incomplete adherence to component 10 (post-administration vital signs check) was due to the prolonged waiting time for pregnant women to receive their scheduled routine antenatal care (ANC) services. For these SHP, ANC service infrastructure and organization were primarily responsible for the lengthy ANC waiting period, thereby making the pregnant women already agitated and exhausted to complete the entire administration process. Consequently, some pregnant women declined further observation of vital signs immediately after administration, considering it a time-consuming and unnecessary step.

“Yes, the issue with this observation is more of time-consuming, i.e., before you send patients for screening, scanning and everything, the time has already gone, and the patient will start complaining, so I think this is why they (SHP) rush through this aspect and finish everything so they can discharge the patient to go home…"*** Research Nurse, Female, Primary Healthcare Facility #2, Kano***

## Discussion

Our study used a mixed-method approach to assess the degree to which SHP adhered to FCM administration. We found that the fidelity level to IV iron implementation was moderate, which varied by facility level (the lowest level attained in PHCs). The most compromised component was ensuring the availability of resuscitation drugs and materials at the point of administration. Our findings showed that teamwork and the availability of charts and protocols contributed to high fidelity. However, institutional / logistical barriers contributed to low to moderate fidelity levels at the facility level.

This moderate level of fidelity in our study was consistent with other quantitative studies and meta-analyses conducted by Durlak et al. [48]. According to the authors, it is impractical to expect complete or near complete adherence as favourable results have been attained at 60%, with few studies reaching above 80% [48]. Additionally, as stated in their review and similar to our study findings, providers in the same study settings have marked variations, usually with differences when comparing the lowest and highest fidelity levels [48]. Their report highlights the diverse levels of fidelity that we observed across different healthcare facility levels in our study (high in tertiary, moderate in secondary and low in PHCs).

It is worth noting that fidelity in most secondary and some primary facilities in our study was high. One facilitation strategy that worked well was collaborative teamwork. Teamwork behaviours exhibited by these SHP were communication of work plans, working in parallel and coordination of FCM administration among team members [49]. The effect of this strategy was consistent with the findings of a study conducted in a real-world setting where an intervention implementation was scaled up across a national healthcare system [50]. It is imperative to note that for the SHP to be fully committed to teamwork as a strategy and to sustain their engagement, it is crucial for the facilities to provide feedback and support at a managerial level as a source of motivation for the SHP [49].

Another strategy that enabled fidelity in our study was the availability and consistent use of protocols and charts, which fostered self-efficacy and, hence, positive participants’ responsiveness, which influenced and enabled the extent to which FCM was administered as intended [30]. Our research findings are similar to the findings from previous studies where program resources and treatment manuals positively influenced their fidelity level [51, 52]. This approach has been proven effective in enabling SHP to apply standardized interventions continuously and accurately to several patients [53].

Fidelity monitoring and feedback are just as critical as facilitation strategies, which have proven effective in overcoming contextual challenges [54, 55] For example, in our study, the use of these strategies overcame the lack of designated space that hindered adherence to ensuring the availability of resuscitation drugs and materials at the point of administration. This approach was similarly effective in previous studies demonstrating the effectiveness of using a standardized strategy to promote high fidelity to EBIs [56, 57] However, despite the availability of designated space, some SHP in some facilities in our study failed to adhere to this component due to their varying perceptions of its delivery. This attitude resulted in low fidelity, which supports Carroll et al.'s statement that more facilitation strategies do not necessarily mean excellent implementation [30]. Although the SHP' attitude resulted in low fidelity, their reasons are practical and realistic, which explains why this component may not be adhered to as intended outside the research-controlled environment. It is of note to acknowledge that adhering to an essential component can become more challenging when implementing EBIs like FCM in real-world settings [58]. On the positive side, the constant availability of and access to resuscitation materials and drugs facilitated emergency readiness before the administration of the intervention, as recommended [36]. However, we must caution that SHP may assume that these resources are always available, even when not, which can lead to life-threatening situations in the event of adverse events.

Unlike the secondary facilities and some PHCs that have a high-fidelity level, a few PHCs in our study had a low to moderate fidelity level primarily due to institutional factors in the process of ANC service delivery. This contextual factor has been linked to insufficient SHP [59]. Generally, the Nigerian healthcare system has one of the lowest health workforce densities (20.1 per 10,000 population), far below the least recommended workforce density of 44.5/10,000 [60]. This shortage of SHP disproportionately affects PHC facilities and the country's northern region, where Kano state is located [61, 62]. This disparity in the HCP-patient ratio led to prolonged patient waiting periods [63–65], resulting in dissatisfaction with the ANC service delivery, mostly in Kano State as observed in our study. Our study found that these prolonged patient waiting periods led to expedited conduct of some essential components and, hence, partial adherence. In line with our findings, it is clear from previous studies that a limited workforce is vital to implementation fidelity, highlighting the need for lasting solutions [66, 67].

Implementing EBIs like FCM in a new setting requires fidelity to the original protocol, but adaptation might also be necessary for the intervention to work and be sustainable [48, 55]. However, controversy still surrounds the balance between fidelity and adaptation [48]. In other words, some authors believe measuring adaptation is necessary when evaluating fidelity [48], while others consider adaptation a lack of implementation success [30, 48]. In our study, the SHP tried to balance following the intervention protocol with adapting it to suit the unique challenges in their facilities. This conduct resulted in some essential intervention components being expedited to address issues like long patient wait times. This expedited conduct was a reactive (adaptation) response to improve the fit of FCM administration to pregnant women [48, 55, 68]. On the other hand, facilities that successfully implemented FCM with high fidelity used planned adaptation strategies, a deliberate attempt to enhance and sustain high fidelity [55]. For instance, the SHP timed the administration of FCM based on workforce capacity and volume of eligible recipients to overcome infrastructural constraints like inadequate human resources and high workload.

## Implications for future practice and research

The findings of our study emphasize the importance of taking practical steps to ensure the high fidelity of FCM administrations in Nigeria's healthcare system. Firstly, it is crucial to introduce strategies to alleviate the excessive workload of SHP, such as health talk-focused videos or AI-powered tools [64, 69]. These tools can assist SHP in educating and counselling pregnant women about their health, including IV iron therapy. Furthermore, it is essential to ensure adequate provision of SHP [65], which could reduce the effect of increased workload, reduce waiting times for pregnant women, and improve their responsiveness to receiving FCM as intended [61, 64]. Additionally, task sharing and task shifting, with sufficient training, should be considered to reduce the impact of FCM administration workload on SHP [70].

Secondly, there is a need to consider the appropriate timing to administer FCM in our day-to-day practice. In our study, we administered FCM during routine ANC services to minimize additional hospital visits to pregnant women outside their scheduled ANC appointments. However, facilities' structure and delivery of ANC services impacted fidelity to protocol. To achieve and sustain high fidelity in real-world settings, we recommend administering FCM on an outpatient basis in specific locations like the lying-in-ward or daycare units [71–74]. Lastly, the SHP need more than an assumption but to be intentional about ascertaining emergency readiness, which is paramount before any IV iron (FCM) administration [36]. Even though the risk of severe adverse reactions to FCM is rare (> 1/10 000 to < 1/1000) [6], SHP need to be aware of the significance of the criticality of such reactions and trained on adequate preparations in case such an eventuality occurs [34].

In our study, we identified the factors that influenced fidelity to FCM in a research setting. Therefore, implementation studies must evaluate adherence to FCM, implement targeted strategies [75] and balance adherence and adaptability in a real-world setting [51]**.** Our checklists may not have accounted for the unique facility-specific or individualized needs and situations surrounding FCM administration. Thus, future studies on similar assessments should consider this factor to promote the scalability and sustainability of IV iron use and fidelity in Nigeria [48, 76]**.**

## Strengths and limitations

To the best of our knowledge, this is the first study in Nigeria to assess and evaluate the fidelity of facilities to IV iron (FCM) administration. We utilized a mixed-method approach through the CFIF guidelines to understand the impact of various factors on the achieved level of fidelity, consistent with the theory of compensatory mechanisms [32]. We established the content validity of our intervention procedure checklist using findings from the literature, including experimental studies, expert agreement and HCP feedback [77]. The triangulation of different data sources improved convergent validity and reliability [78]. Due to this study's pragmatic conduct, we had no control over how the ANC services are delivered in day-to-day practice. However, the intervention was administered in a supplemented environment, which might not have accurately reflected the actual needs relevant to the fidelity of FCM in the present Nigeria healthcare system. Nevertheless, our detailed report enhanced trustworthiness and ease of replicability for future research in similar settings [33, 77]. There is the possibility that the SHP in facilities with high fidelity might have changed their behaviour because they are aware of being observed [79] However, it is unlikely that their awareness influenced the fidelity level throughout the study period due to institutional challenges [33, 78]. Furthermore, because we observed every other FCM administration, SHP may become habituated and less likely to notice being observed. Additionally, there is the possibility of bias in our data collection method [80], the risk of which was reduced by engaging skilled and neutral research assistants.

## Conclusions

Our study aimed to evaluate SHP adherence to implementing FCM and identify the factors that influenced this adherence. Our findings revealed that while fidelity levels were moderate generally, they varied across facility types. However, some PHCs, all secondary and tertiary centres that effectively utilized facilitation strategies, had high fidelity. Unfortunately, a few PHCs had low adherence due to intervention complexities and contextual factors. To ensure high fidelity, targeted strategies are necessary, including providing adequate SHP to reduce heavy workloads and patient waiting times and ultimately improve FCM implementation quality. Further research conducted in a real-world setting could help identify and test these facilitation strategies and improve healthcare facilities' adaptability.

### Supplementary Information

Supplementary Material 1.

Supplementary Material 2.

Supplementary Material 3.

Supplementary Material 4.

## Data Availability

The datasets utilized and/or analyzed during the index study will be made available by the corresponding author upon request.

## Abbreviations

FCM: Ferric carboxymaltose
SHP: Skilled health personnel
PHC: Primary health centres
IDA: Iron deficiency anaemia
AIP: Anaemia in pregnancy
IV: Intravenous
pH: Potential of hydrogen
Hb: Haemoglobin
HICs: High income countries
LMICs: Low and middle-income countries
IVON-IV: Versus oral iron for Iron Deficiency Anaemia in Pregnant Nigerian Women
StaRI: Standards for Reporting Implementation Studies
CFIF: Conceptual framework for implementation fidelity
SOP: Standard operating procedure
REDCap: Research Electronic Data Capture
IDIs: In-depth interviews
IC: Intervention complexities
FS: Facilitation strategies
QA: Quality of administration
PR: Participants responsiveness
C: Context
IVF: Intravenous fluid
IQR: Interquartile range
ANC: Antenatal care
