# Supplementary material for: Implementation fidelity of intravenous ferric carboxymaltose administration for iron deficiency anaemia in pregnancy: a mixed-methods study nested in a clinical trial in Nigeria
Source: Implement Sci Commun. 2024 Jul 23;5:81. doi: 10.1186/s43058-024-00609-5 (PMC11264421; doi:10.1186/s43058-024-00609-5)
Supplement: Supplementary file 2 — Supplementary Material 2. [file 43058_2024_609_MOESM2_ESM.docx]

**ALGORITHM FOR PROCEDURE OF THE INFUSION OF IV FERRIC CARBOXYMALTOSE**

Study Drug and Infusion Set Inventory

- IV ferric carboxymaltose
- 100 ml bottle of 0.9% normal saline
- Pink cannulae/butterfly set
- IV fluid infusion set,
- Needles and syringes

Resuscitation Medication Inventory

- Adrenaline, IV chlorpheniramine,
- IV ranitidine, Oral ranitidine
- IV hydrocortisone
- 500mls or 1 litre bottle normal saline
- IV fluid infusion set
- Oxygen mask, Oxygen cylinder

1. Check availability of the following resuscitation medications and materials in the procedure room

2. Patient Counseling

- Confirm patient’s randomization group is FCM (ferric carboxymaltose group)
- Obtain verbal consent for IV iron administration from participant
- Complete patient counseling (see IVON Patient Counseling Script)

3. Drug Administration

- Perform and document vital signs: PR, BP, SPO2, RR

- Calculate and document the maximum individual dose at 20 mg/kg body weight up to a maximum dose of 1000 mg.

- Withdraw the equivalent volume of the calculated dose from the 20 mL vial of ferric carboxymaltose.

- Dispense into the 200 ml 0.9% normal saline bottle.

-Prepare patient and get a good venous access from a large vein, preferably antecubital fossa.

- Set up the administration at low flow rate of 100 drops per minute.

- Observe patient closely and monitor and document pulse rate, blood pressure, and respiratory rate for the first 2 minutes.

- If the patient does not experience any reaction, increase the flow rate, and complete the IV iron infusion in 20 mins.

4. Post drug administration

- Post-administration vital signs check

-Patient is observed for adverse effects for at least 30 minutes following completion of

IV iron infusion.

-Document procedure in the patient’s case note
