# Supplementary material for: Implementation fidelity of intravenous ferric carboxymaltose administration for iron deficiency anaemia in pregnancy: a mixed-methods study nested in a clinical trial in Nigeria
Source: Implement Sci Commun. 2024 Jul 23;5:81. doi: 10.1186/s43058-024-00609-5 (PMC11264421; doi:10.1186/s43058-024-00609-5)
Supplement: Supplementary file 3 — Supplementary Material 3. [file 43058_2024_609_MOESM3_ESM.docx]

**SITE ID/ Centre**

**Study ID**_________________________________

**Date**_________________________________

**Intervention Procedure Checklist for Intravenous Ferric Carboxymaltose Administration (FCM)**

**Critical Steps to IV FCM Administration**

| **Procedure** | **Done Partially done Not done** |
| --- | --- |
| 1. Ensure the following resuscitation medications and materials are available at the point of administration:  i. Adrenaline  ii. IV Chlorpheniramine  iii. IV Ranitidine  iv. Oral Ranitidine  v. Intravenous hydrocortisone  vi. 500mls/1L bottle of Normal saline  vii. Intravenous fluid giving set  viii. Oxygen mask  ix. Oxygen cylinder | **✓ ✓** |
| 2. Ensure the availability of FCM | **✓ ✓** |
|  |  |
| 3. Patient Counselling | **✓ ✓ ✓** |
|  |  |
| 4. Confirm patient's randomization group is FCM | **✓ ✓** |
|  |  |
| 5. Obtain verbal consent for FCM administration from Participant | **✓ ✓ ✓** |
|  |  |
| 6. Perform baseline vital signs: Respiratory rate (RR), Oxygen  Saturation (SP02), Pulse rate (PR), Blood pressure (BP) | **✓ ✓ ✓** |
|  |  |
| 7. Calculation of the individual dose at 20mg/Kg body weight up to a  maximum dose of 1000mg | **✓ ✓** |
| 8. Set up the administration at a low flow rate | **✓ ✓ ✓** |
|  |  |
| 9. Observe the patient closely and monitor RR, SP02, PR, and BP for  the first 2 minutes | **✓ ✓ ✓** |
|  |  |
| 10. Post-administration vital signs check | **✓ ✓ ✓** |
