# Supplementary material for: Implementation fidelity of intravenous ferric carboxymaltose administration for iron deficiency anaemia in pregnancy: a mixed-methods study nested in a clinical trial in Nigeria
Source: Implement Sci Commun. 2024 Jul 23;5:81. doi: 10.1186/s43058-024-00609-5 (PMC11264421; doi:10.1186/s43058-024-00609-5)
Supplement: Supplementary file 4 — Supplementary Material 4. [file 43058_2024_609_MOESM4_ESM.docx]

|  | **Purpose** | The purpose of this interview is to understand factors that influenced the adherence of facilities through the SHPs to the intervention protocol.  **Study in-depth interview guide for skilled health personnel**  **Study Title**: Implementation fidelity of intravenous ferric carboxymaltose administration for iron deficiency anaemia in pregnancy: A mixed-methods study nested in a clinical trial in Nigeria |
| --- | --- | --- |
|  | **Guidance to the interviewer** | *Before the interview:*   1. Before beginning the interview, introduce yourself and explain your role in the study 2. Read the verbal consent script to potential participants (oral consent script form). 3. Encourage the potential participant to ask any clarifying questions and check for understanding. 4. Ask for and confirm verbal permission to interview the participant. If they are hesitant or say no, do not go ahead with the interview. 5. Collect all socio-demographic data BEFORE interviewing consented participants using the questions on the socio-demographic form. 6. If there are no further questions, take permission to record and begin the discussion. (Begin recording)   *During the interview:*   1. Note that the questions below are open- ended and semi-structured in nature with room to add more questions based on the SHPs' responses for better clarity or to expand upon areas that require further explanation.   *End of the interview:*   1. Ask participants if they have anything else to say about the topic that was not asked during the interview. 2. Summarize the discussion and assess accuracy from participants. 3. Thank the participants for their time. 4. Answer any remaining questions. 5. Put off the recorder 6. Provide contact information if they have any questions or concerns: |

| **Icebreaker** |
| --- |
| **To participants:** Please introduce yourself and what nickname you would like to be called by, in this discussion?  Describe your role in the administration of IV iron. |

| **Title of each section with corresponding**  **influencing factors** | **Main questions** | **Probe** |
| --- | --- | --- |
| A. SHP’s experience of  administering FCM  Participant’s responsiveness  Intervention complexities | What is your experience with administering FCM to pregnant women in your facility?  Reflect on the aspects of the administration of FCM you find easy to implement.  How about the aspect of the administration process you found challenging to implement? | - Please describe your process of administering FCM to a pregnant woman. - Please discuss why you find this aspect easy to implement? - Kindly discuss why you find this aspect challenging? |
| B. Views on the resources and  training to FCM according to  the protocol  Facilitation strategies  Quality of delivery | What were the things that enabled you to administer FCM safely to the  pregnant women?    What support tools were available for you as a guide to administer FCM according to the protocol?  **Prompt:** You may relate your answers to the resources provided as support tools for safe administration, such as training materials, SOPs, and algorithms to serve as a guide on the step-to-step process in the administration of IV iron, frequent feedback on performance etc.  How many trainings did you receive before and during the trial? | - Please describe how you used these tools to administer FCM. - How often did you use these support tools? - What were the effects of these support tools on your administration of FCM? - Please reflect on the training received before and during the trial and discuss your experience with the training. - Describe how it has helped you to administer FCM according to the protocol. - What were the things that were not helpful about the training? |
| C. Challenges and barriers to administering FCM according to the protocol  Intervention complexities  Context | What were the barriers you encountered while administering FCM?  **Prompt:** You can discuss these barriers regarding the suitability of the place where FCM is administered, time constraints, too many patients, limited staff, health worker’s attitudes etc.  Focusing on the algorithm (step-by-step bullets guide) provided as a guide to administer FCM, which of the steps of the algorithm did you have challenges implementing while administering FCM?  **Instruction:** Please read through all the steps (components) of the algorithm to enable the participant’s recollection of each step of the FCM administration.  Some of the facilities had challenges with some steps of FCM administration. For example, some had challenges with step 1 (ensuring the availability of resuscitation drugs and materials), step 3 (patient counselling), step 6 (performing baseline vital signs) and step 10 (post-administration vital signs check). So, we want to hear your opinion on the challenges they might have encountered while administering FCM starting from the 1st step. Why do you think those facilities have challenges with step 1? What about step 3? Regarding step 6, what is your view on the challenges they had carrying out this step as per protocol? What about step 10?  **Specific questions for facilities with low fidelity level:**  We know you are skilled and trained to give FCM which you administered safely, and you have all the resources available to administer it safely. However, we noticed you had challenges implementing steps 1 and 10 of the FCM algorithm chart. What were the challenges you encountered?  **Prompt**: the challenges may be facility based, patient based, personal factors etc.  **Specific questions for facilities with high fidelity level**: What were the challenges you experienced while administering FCM to pregnant women?  **Prompt:** this may be in terms of workload, low staff strength, patient’s time etc. | - What were the effects of the barriers on your administration of FCM? - Why was/ were these step/steps challenging? (You can give an example or discuss where you have these challenge(s)) - Why do you think those facilities have challenges with step 1? - What about step 3? - Regarding step 6, what is your view on the challenges they had   carrying out this step as per protocol?   - What about step 10? - Please can relate your responses to the experiences you had and to the peculiarities in your site as well. - Why did you have challenges with those steps? Please elaborate on those challenges with instances on when they occur - Please elaborate on those challenges with instances on when they occur |
| D. Strategies to overcome the challenges to administering FCM as per protocol    Facilitation strategies Quality of delivery | **Questions for all facilities**: Based on all the challenges and peculiarities we have discussed, what strategies would you suggest for administering FCM according to the protocol in the future?  **Questions for all facilities with high fidelity level:** We discovered that you had a high-adherence level all through the period of this trial, how were you able to achieve this (i.e., administer FCM according to the protocol) despite the challenges and the peculiarities in your site? | • Why do you think this strategy is the right fit for this challenge?  • What were the strategies that enabled you to overcome the  challenges?  • What were the effects of the strategies implemented to solve  those challenges? |
